# Integral strategy to supportive care in breast cancer survivors through occupational therapy and a m-health system: design of a randomized clinical trial

**DOI:** 10.1186/s12911-016-0394-0

**Published:** 2016-11-25

**Authors:** Mario Lozano-Lozano, Lydia Martín-Martín, Noelia Galiano-Castillo, Francisco Álvarez-Salvago, Irene Cantarero-Villanueva, Carolina Fernández-Lao, Carmen Sánchez-Salado, Manuel Arroyo-Morales

**Affiliations:** 1 Department Physical Therapy, University of Granada, Granada, Spain; 2 Mixed University Sport and Health Institute (iMUDS), Granada, Spain; 3 Institute for Biomedical Research ibs.GRANADA, University Hospital Complex of Granada/ University of Granada, Granada, Spain; 4 Breast Oncology Unit, Virgen de las Nieves Hospital, Granada, Spain

**Keywords:** Breast, Neoplasms, Occupational therapy, Mobile applications, Quality of life

## Abstract

**Background:**

Technological support using e-health mobile applications (m-health) is a promising strategy to improve the adherence to healthy lifestyles in breast cancer survivors (excess in energy intake or low physical activity are determinants of the risk of recurrence, second cancers and cancer mortality). Moreover, cancer rehabilitation programs supervised by health professionals are needed due to the inherent characteristics of these breast cancer patients. Our main objective is to compare the clinical efficacy of a m-health lifestyle intervention system alone versus an integral strategy to improve Quality of Life in breast cancer survivors.

**Methods:**

This therapeutic superiority study will use a two-arm, assessor blinded parallel RCT design. Women will be eligible if: they are diagnosed of stage I, II or III-A breast cancer; are between 25 and 75 years old; have a Body Mass Index > 25 kg/m^2^; they have basic ability to use mobile apps; they had completed adjuvant therapy except for hormone therapy; and they have some functional shoulder limitations. Participants will be randomized to one of the following groups: integral group will use a mobile application (BENECA APP) and will receive a face-to-face rehabilitation (8-weeks); m-health group will use the BENECA app for 2-months and will received usual care information. Study endpoints will be assessed after 8 weeks and 6 months. The primary outcome will be Quality of Life measured by The European Organization for Research and Treatment of Cancer Quality of Life Questionnaire Core and breast module. The secondary outcomes: body composition; upper-body functionality (handgrip, Disability of the Arm, Shoulder and Hand questionnaire, goniometry); cognitive function (Wechsler Adult Intelligence Scale, Trail Making Test); anxiety and depression (Hospital Anxiety and Depression Scale); physical fitness (Short version of the Minnesota Leisure Time Physical Activity Questionnaire, Self-Efficacy Scale for Physical Activity); accelerometry and lymphedema.

**Discussion:**

This study has been designed to seek to address the new needs for support and treatment of breast cancer survivors, reflecting the emerging need to merge new low cost treatment options with much-needed involvement of health professionals in this type of patients.

**Trial registration:**

ClinicalTrials.gov Identifier: NCT02817724 (date of registration: 22/06/2016).

## Background

Cancer is one of the most incident diseases worldwide. Over 14 million new cancer cases occur every year, but are projected to reach approximately 22 million by 2030 [1]. Among all cancer types, breast cancer is the most commonly diagnosed cancer in women, and approximately 4.4 million women worldwide live with a diagnosis of breast cancer [2]. Fortunately, the survival rate is very encouraging; the estimated number of deaths from breast cancer is estimated to be less than one-third of new cases [1]. Developments in screening and improved treatments for breast cancer have led to improved survival, and it is beginning to be regarded as a chronic disease [3, 4]. This new perspective of the disease has led to a growing need for long-term treatments [4] with an integrative strategy that takes into account the patients’ lifestyles and physical, cognitive and emotional impairments [5–7].

Regarding the importance of the patients’ lifestyles (in physical activity and diet), the literature highlights the importance of maintaining healthy lifestyles to reduce the risk of recurrence, secondary cancers or death. There is strong evidence about the efficacy and safety of exercise and healthy diet to improve the patients’ quality of life (QoL) [8, 9] and reduce the effects of cancer [10, 11]. Recent research reveals that even when patients know the benefits of interventions aimed at promoting energy balance among cancer survivors (in terms of intake and physical activity), it is unrealistic to expect that most of them, who have a strong sedentary habit, will comply with the current good practice guidelines [10, 12]. In addition, survivors report difficulties in adhering to and maintaining an appropriate lifestyle [12]. Consequently, this energy imbalance increases the risk of cancer recurrence [13] and, along with the functional limitations and emotional/occupational imbalance, reduces the QoL of breast cancer survivors (BCS) [14]. New strategies with a comprehensive approach of support must be developed to improve the adherence and motivation of these patients and to reduce the high cost involved in creating individualized exercise programs and diets [15]. Currently, technological support is a promising strategy that could improve issues, such as barriers of distance, time, cost and motivational aspects [16]. Telehealth systems, which are based on computers and mobile applications (m-health), offer a promising approach for both dietary and physical activity assessments [17] and the patients’ motivation can be significantly increased through the immediate feedback provided by these systems [18]. A recent study has developed a mobile application to simultaneously collect data on diet and physical activity in adults [17], but, to our knowledge, no programs exist that simultaneously collect data on diet and physical activity in cancer patients and provide immediate feedback with individualized recommendations.

In addition to the need to improve these patients’ lifestyles, the patients may experience physical, cognitive and emotional impairments. The most common upper body symptoms reported by BCS are related to shoulder impairments [19–21], although much research has supported the practice of performing early exercises to avoid limitations of range of motion (ROM) in the shoulder [22, 23]. Moreover, cognitive impairment occurs in 10%–50% of these women [24, 25], and the emotional distress caused by shifts in social support and the fear of recurrence and death has also impacted women’s wellbeing [26, 27]. The performance of daily tasks (such as activities in daily living, work, and leisure tasks) are influence by all these complications and, along with unhealthy lifestyle habits, affect the overall QoL [22].

In this sense, occupational therapy is an effective intervention to improve the patients’ QoL, ROM or distress in different conditions [28–30]. However, to our knowledge, the only published randomized controlled trial evaluating occupational therapy in BCS is an intervention aimed at reducing the limitations of rural patients in their daily activities [31]. The authors found that a telephone-based problem-solving occupational therapy intervention program was feasible and had positive effects on the patients’ function, QoL and emotional state. However, the study had methodological limitations, such as a small simple size and intervention bias. Other previous studies with the aim of evaluating the effects of occupational therapy on cancer patients had several limitations, such as including any type of cancer [32–36], the use of a non-randomized controlled trial (RCT) approach [33–35, 37, 38], and pilot studies [31, 36] involving very few patients [31, 33, 36–38].

This study arises from the need to establish an integrative and multidisciplinary strategy to support BCS by taking advantage of the features of these two proposals: first, the functionality and independence provided by a mobile application that patients can use when and wherever they choose; secondly, the imperative need for a supervised face-to-face intervention by a health professional, due to the inherent characteristics of these patients. Our study aims to compare the clinical efficacy of an m-health lifestyle intervention system alone versus an integrative strategy that also includes a face-to-face intervention in BCS. In this manuscript, we describe the design and methods of the study.

## Methods

### Objectives

The main objective of this RCT is to assess if an integrated strategy that uses an m-health system in addition to a face-to-face treatment is better than the use of the m-health system alone to improve the immediate and long-term QoL of BCS. Second, we want to examine the effects of the interventions on the overall impact on functionality, body composition, anxiety and depression, physical measurement, lymphedema and cognitive function. The integral group will use the m-health and receive three occupational therapy sessions each week for an 8-week period. We will also study the effect of a 24-week period without rehabilitation on the studied variables. We hypothesize that support care based on an Occupational Therapy-supervised rehabilitation program will promote functionality and the combination with the mobile system will improve the patients’ lifestyles and QoL, reduce distress, and improve cognitive function and arm mobility.

### Research design and methods

The present study is a parallel group, assessor-blind, superiority RCT that will be conducted using assessments at baseline and immediately after the 8-weeks intervention. Follow-up measurements will be collected for 24 weeks after the end of the 8-week intervention period, resulting in a total trial data collection period of 32 weeks. We will use two separate assessment days to avoid fatigue in patients. In Table 1 is shown the study assessment schedule.

**Table 1:** Study assessment schedule

Assessment	Baseline	Post-intervention (8 weeks)	Follow – up (24 weeks)
Informed Consent	x		
Day 1 testing			
Sociodemographic data	x		
Anthropometric data, pressure/rate heart	x	x	x
Accelerometry (1 week)	x	x	x
Body Composition (DEXA)	x	x	x
Minnesota	x	x	x
Self-efficacy Physical Activity questionnaire	x	x	x
EORTC QLQ-C30	x	x	x
EORT QLQ-BR23	x	x	x
Day 2 testing			
WAIS-IV^a^(subtest)	x	x	x
Trail Making Test (TMT)	x	x	x
Handgrip strength	x	x	x
Lymphedema	x	x	x
Hospital Anxiety and Depression Scale	x	x	x
DASH	x	x	x
Goniometry	x	x	x

^a^Working memory and processing speed subtest

### Participants

A total of 80 eligible (see inclusion criteria below) BCS will be randomized into the integral group (*N* = 40) or the m-health group (*N* = 40). For feasibility, the study is conducted in three waves. During the first year of the study (from January to December 2016), we will prepare protocols, establish the measurement techniques, and enrol the first 25 women in the study. At the beginning of the second year of the study (between January and April 2017), we will enrol an additional 30 women, and in the third stage, we will enrol the remaining 25 women. In summary, the target sample size of 80 BCS will be achieved in these 3 waves.

The integral group will receive the m-health plus an 8-week occupational therapy onsite program, and the m-health group will only use the app. Participants will be enrolled in this study by oncologists from the Hospital Virgen de las Nieves (Breast Unit) and the Hospital Clínico San Cecilio, Granada (Spain). The Research Ethics Committee of the province of Granada approved this study.

### Eligibility criteria

Eligible women require: 1) to be between 25.0 and 74.9 years-old, 2) to be diagnosed of stage I, II or IIIA breast cancer, 3) to have medical clearance of participation, 4) to be overweight or obese, according to the *Spanish Society for the Study of Obesity* (SEEDO) [39], 5) to have basic ability to use mobile apps or living with someone who has this ability, 6) completion of adjuvant therapy except for hormone therapy, 7) to have some functional or ROM limitations measures by goniometry and the Disabilities of the Arm, Shoulder and Hand (DASH) questionnaire, and 8) to have signed informed consent and have interest in improving lifestyle.

The exclusion criteria were defined as follows: history of cancer recurrence, to have had chronic disease or orthopaedic issues that would interfere with ability to participate in this rehabilitation program, or to have had uncontrolled hypertension (diastolic pressure > 95 mm Hg).

### Outcome measures

The primary outcome measure is QoL. The secondary outcome variables include body composition, active range of motion (AROM), functionality, anxiety and depression, and cognitive function. Other variables of interest include muscular strength and free-living physical activity.

### Primary outcome measure

Quality of life: *The European Organization for Research and Treatment of Cancer Quality of Life Questionnaire Core 30* (EORTC QLQ-C30) version 3.0 [40]: We will use the EORT QLQ-C30 to assess QoL. This questionnaire is one of the most widely used instruments to measure QoL in cancer patients. The QLQ-C30 is composed of both multi-item scales and single-item measures, as well as five functional scales, three symptom scales, a global health status/QoL scale, and six single items. The scores must be averaged and linearly transformed to obtain a range of scores from 0 to 100, with a higher score representing a greater response level. Thus, a high score for a functional scale represents a healthy level of functioning and a high score for the global health status represents a high QoL, but a high score for the symptom scale represents a high level of symptomatology [41]. The test/retest reliability is high for all scales, ranging from 0.82 to 0.91 [40].

*The European Organization for Research and Treatment of Cancer Breast Cancer-Specific Quality of Life Questionnaire* (EORT QLQ-BR23) [42]: This questionnaire is a breast cancer module of the EORTC QLQ-C30 that contains 23 items rated on a four-point scale ranging from 1 (not at all) to 4 (very much). The items assess the side effects of therapy, arm symptoms, breast symptoms, body image, and sexual function. Additionally, there are single items assessing sexual enjoyment, anxiety caused by hair loss, and future outlook. The scores range between 0–100 points. The procedure for scoring the breast cancer module is the same as the EORTC QLQ-C30 [41]. For scales evaluating function, a higher score represents a higher level of functioning. For scales evaluating symptoms, a higher score indicates more severe symptoms. The reliability has been shown to be high to moderate (Cronbach’s α ranged between 0.46 – 0.94) [42].

### Other outcome measures

#### Body composition

Height and weight will be measured. Body mass index, fat mass, lean body mass, abdominal adipose tissue and bone mineral density will also be assessed by conducting Dual-energy X-ray absorptiometry (DXA, Discovery densitometer from HOLOGIC, QDR 4500 W) using protocols reported in previous studies [43, 44]. This assessment tool has previously been used in breast cancer patients [45, 46].

### Muscular strength

The handgrip strength test will be assessed using a digital dynamometer (TKK 5101 Grip-D; Takey, Tokyo, Japan). Following the protocol described by Ruiz-Ruiz et al. [47], the optimal grip span will be determined by a simple algorithm to adapt the dynamometer. Throughout the whole test, BCS will be in a bipedal position; they have to put their arm in complete extension without touching any part of their body [18], repeating the test three times with each hand, alternately. There will be a delay of one minute between each test. The mean of the three tests will be used for the main analysis. This measurement has been demonstrated to be valid and reliable [48].

### Upper body functionality

The disability of the arm, shoulder and hand (DASH) questionnaire: the American Academy of Orthopedic Surgeons introduced the DASH questionnaire as a specific instrument to measure upper extremity functionality [49]. It is one of the most extensively used tools [50]. Of the 30-items that are included in the DASH questionnaire: 21 items ask about the degree of difficulty in physical activities; 5 items ask about the severity of some pain symptoms; and the final 4 items ask about other activities such as social activities, sleep, work or self-image. The impact of the symptoms on each activity is also assessed. The scale score ranges from 0 to 100 points; the higher the score, the greater the disability [51]. The reliability of the Spanish version has a Cronbach’s α = 0.96 [52].

### Active range of motion (AROM)

Shoulder AROM measurements will be obtained using a standard, two-armed goniometer, which is described as the clinical gold standard [53]. The patients will be asked to actively move their arms as much as they can to obtain measurements (in degrees) of flexion, extension, abduction, internal rotation and external rotation of the shoulder [23]. The movement will be validated by the interviewer and motion compensation will be limited to avoid overestimating the scores.

### Cognitive function

Wechsler Adult Intelligence Scale (WAIS-IV): The WAIS-IV is an intelligence test designed to measure cognitive ability in adults and older adolescents and provides the most advanced adult measure of cognitive ability [54]. WAIS-IV subtests will be administered and scored according to standardized procedures [55]. For feasibility issues and because specific subtests provide information on a specific cognitive function (and can be used separately [55]), we will use two of the four index scores that compose the test: the Working Memory Index (WMI) and the Processing Speed Index (PSI). The WMI includes two subtests, Arithmetic and Digit Span, and the PSI also includes two subtests, Digit Symbol-Coding and Symbol Search.

The Trail Making Test (TMT) measures the flexibility of thinking using a visual-motor sequencing task and is one of the most important neuropsychological tests, providing information on speed of processing, visual search, mental flexibility, scanning and executive functions [56]. It is formed by two subtests. TMT-A requires the participant to draw lines that sequentially connect several encircled numbers (1 to 25) distributed on a sheet of paper. TMT-B is similar in requirements, but in this case, the participant must alternate between numbers and letters (e.g., 1, A, 2, B, 3, C, etc.). The score is based on the amount of time required to complete the task.

### Anxiety and depression

The Hospital Anxiety and Depression Scale (HADS): This scale consists of 14 items with two subscales (seven items for anxiety and seven for depression) and a score which ranges from 0 to 21 for each subscale. The questionnaire contemplates a cutoff point of 11 or above to consider anxiety and depression conditions [57, 58].

### Physical fitness

Short Version of the Minnesota Leisure Time Physical Activity Questionnaire (VREM) [59]: This questionnaire is a short version of the original Minnesota questionnaire [60] and is composed of 5 items. It asks for the period in a typical week that the participants perform routine housework activities (cleaning house and go shopping on foot). In addition, it asks about activities performed during the last month or in a typical month for the other items, such as walking, working in the garden, playing sports or dancing and climbing stairs. Finally, energy expenditure is calculated (in METS-min/14 days) and the participant is classified from sedentary to very active according to their energy expenditure [59].

Self-Efficacy Scale for Physical Activity (EAF): The EAF is a validated instrument that determines the participants’ beliefs about their own abilities to perform physical activities (self-efficacy for physical activity). It also allows us to identify the barriers and limitations that prevent the user from practicing this behaviour and the strength they require to perform regular physical activity. The EAF consists of three domains: scheduled physical exercise, physical activity in daily activities and walking. A total of 39 items are rated from 0 to 10; the higher the score, the greater the ability to perform the activity [61, 62].

### Accelerometry

Accelerometry will be used to obtain data about physical activity and sedentary time for each participant, following a previously published protocol for usage and analysis [63]. A pre-programmed tri-axial accelerometer (ActiGraph GT3X+, Pensacola, Fl., US) and a daily questionnaire will be given to BCS. The participants will wear the accelerometer for 8 consecutive days. They will be instructed to wear the accelerometer on their lower back for the whole day (including when sleeping) but to take it off during aquatic activities. They will also receive an information sheet with detailed instructions. Participants will be included in the main analysis if the device records data for at least 4 days over a period of at least 10 h each day. Data will be collected at intervals of 1 min. Nonwear periods (intervals of 60 consecutive minutes with zero counts) and the first day of wearing the device will be excluded from analyses. Accelerometer data will be downloaded to the same computer used to initialize them [18].

### Lymphedema

We will measure changes in size or volume of the upper limbs to diagnose lymphedema. An inextensible flexible tape 0.5 cm wide x 2 m long with an accuracy of 0.1 cm will be used following the protocol using in some previous studies [18, 64], which has been shown to be valid and reliable [65, 66].

### Sample size

**Fig. 1:**
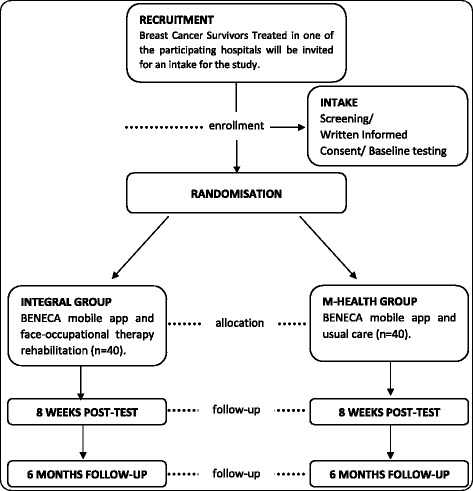
Flow diagram showing the recruitment of patients

The sample size and power calculations for this trial were obtained through overall Health-Related QoL (HRQoL) using EORTC QLQ-C30 version 3.0 [40], and taking into account previously reported data [67] a minimally important difference from 5 to 10 points was considered. Assuming that integral group increase HRQoL in BCS in compared with m-health group [18] we can detect differences of at least 5% with a power of 90% and an α of 0.05 with two groups (Integral group and m-health group) of 36 participants assuming similar standard deviation (approximately 7 points). A maximum loss at follow-up of 10% will be allowed to face a possible drop-out rate [9]. Hence, we will recruit 80 BCS (40 in each group). Fig. 1 shows the flow diagram of the study participants.

### Randomization and blinding

To reduce the risk of bias during the assessment, after completion of the baseline assessment we will allocate eligible patients randomly either m-health or integral groups into three randomization waves, using computer-generated numbers (EPIDAT 3.1, Xunta de Galicia). An external member will introduce the sequence in sealed opaque envelopes. Assessment staff will be blinded to patients’ randomization assignment and the staff responsible of the rehabilitation program will not be able to change any assignment. After the 6-month follow-up period, and because of ethical implications, once the last outcome variable has been measured we will invite participants of the m-health group to participate into the face-to-face rehabilitation program.

### Integral group

The intervention will be implemented by the CUIDATE research group. The supervised face-to-face program involves two parts (8 weeks in total). The participants will may use the BENECA System (mobile app) daily, which aims to recover a healthy lifestyle in BCS (in terms of energy balance: physical activity and dietary). On the second day of the assessment, staff will install the app on the participants’ mobile phones and will train them to use it. Furthermore, the women will receive telephone calls and text messages (as required) to resolve any questions and provide suggestions, and a video tutorial on how to use the application is also available on the web.

Moreover, participant will receive a supervised face-to-face rehabilitation program. This intervention consists of a supervised-occupational therapy rehabilitation program at the iMUDS centre (Mixed Institute University Sport and Health). Because there is no information about the ideal occupational therapy program for breast cancer patients, we have developed a comprehensive program that covers most of the physical, cognitive and emotional needs of these patients after oncology treatment from the occupational therapy perspective.

The second part is based on the fact that this span has been used in previous RCTs that have similar outcomes and samples [9, 18]. The occupational therapy program includes 3 weekly sessions of 60 to 90 min each. The weekly sessions will be distributed as follows: 2 sessions/week in a ROM-cognitive subprogram (approximately 50 min/session) using therapeutic workshops and individualized treatment that focus on improving the ROM, muscle strength and endurance, and manipulative skill and energy conservation as well as cognitive activities; 3 sessions/week of a psychomotricity program (approximately 45 min/session) including activities to improve functional capacity and address fatigue and pain as well as a warm-up period and relaxation techniques; and finally, 1 session/week of a psychosocial intervention (approximately 30 min/session), working on areas of ergonomics, techniques of energy conservation and fatigue management, job anxiety, coping techniques and occupational balance. All of these exercises will be assigned to women in the integral group according to their perceived needs at the baseline assessment. These needs will be established based on the fatigue levels, pain, functional capacity, ROM, and distress levels reported by the patients. Therefore, each participant will receive individual and progressive training (for example, the number and type of exercises, series, repetitions and so on). Efforts will be made to prevent the integral group from receiving additional physical care.

### M-HEALT: BENECA APP system

BENECA asks users to register their food and drinks and the different activities performed during the previous day. With an open structure and four time periods, the application will take the form of a questionnaire on the diet (over the last 24 h) and a record of daily activities in terms of duration and intensity. Users also record their weight (kg) and height (cm). After entering the information, the system will provide the patient information about their energy balance and general recommendations on physical activity according to their individual profile, using the reference guide for exercise in cancer patients from the American College of Sports Medicine [68]. Additionally, it provides recommended substitutions for foods that are considered potentially carcinogenic with others that may have a protective capacity against cancer, according to the guidelines of the American Cancer Society [11, 69] and the recommendations of the WCRF about the consumption of food of plant and animal origins, food with low energy density, etc. Furthermore, the program also detects the presence of an energy imbalance.

### Telephone calls

The CUIDATE group will make the telephone calls and send messages of encouragement. On the one hand, with this calls, participants will be able to solve any problems with the usage of BENECA app. Moreover, we will check the patients’ improvement and satisfaction. On the other hand, the aim of messages will be to stimulate not only the adherence with BENECA app but also with the program.

### M-Health group

Because it is a study of therapeutic superiority, the m-health group will use the BENECA app for 2 months and will receive some general recommendations about healthy lifestyle, stress management and occupational balance in paper format. After completion of this study, the m-health participants will be given the opportunity to participate in the supervised face-to-face program due to the ethical concerns of the CUIDATE group. The data obtained will be not used in this study.

### Data analysis

All analyses will be carried out using STATA/SE 14.0 StataCorp, College Station, TX, USA) or using Statistical Program for Social Sciences (IBM© SPSS© Statistic version 20, Corp., Armonk, NY). We will check the normal distribution of variables with Kolmogorov-Smirnov and Shapiro-Wilk test, as appropriate, and the differences at baseline between groups with Chi-square test or Student *t*-test, as appropriate. The main analysis will be repeated measures analyses of the covariance (ANCOVA) with age, type of surgery, tumour stage and time since diagnosis as covariates. Intergroup effect sizes will be calculated to provide change magnitude information. We will use the intention-to-treat principle for all analyses.

## Discussion

This RCT will investigate whether there are clinically relevant differences in improvements in the QoL of BCS between an integral strategy and the use of the m-health system alone. This study has been designed to address the new needs for support and treatment of breast cancer survivors, reflecting the emerging need to merge new, low cost treatment options with the much-needed involvement of health professionals in the treatment of this type of patients. The supervised program includes not only strengthening and range of motion exercises of the shoulder, which are necessary in these patients [9], but also features a cognitive [25] and psychosocial [27] approach in a single intervention program which, together with the use of the m-health application [17], provides the integral character of the project.

In addition, most studies in cancer patients have been conducted with a rehabilitation team comprising nurses, psychologists and physiotherapists [8, 27]. For this reason, we chose to use a supervised face-to-face rehabilitation program conducted by an occupational therapist, due to the holistic and integrative approach of the discipline. Although we expect to see improvements in the primary outcome in both groups, we hypothesize that the combination of the supervised program and the m-health system will cause significant differences in QoL compared with the m-health group. QoL improvement is considered an indicator of cancer rehabilitation success [70]. If this integral option is effective, it will highlight the need for health systems to include disciplines such as occupational therapy in the supportive care of cancer patients during the survival period, as well as the potential advantage and cost reduction provided using a mobile app. Moreover, the results of this study could garner support for the use of this type of strategy in an increasing number of 17.8 million cancer patients in the European Union [71], with a high proportion of them claiming adequate rehabilitation services.

## Abbreviations

App: Mobile application
AROM: Active range of motion
BCS: Breast cancer survivors
DASH: Disabilities of the arm, shoulder and hand
EORT QLQ-BR23: European Organization for Research and Treatment of Cancer Breast Cancer-Specific Quality of Life Questionnaire
EORTC QLQ-C30: European Organization for Research and Treatment of Cancer Quality of Life Questionnaire Core (30)
HADS: Hospital anxiety and depression scale
HRQoL: Health-related quality of life
m-Health: Mobile health application
QoL: Quality of life
RCT: Randomized controlled trial
ROM: Range of motion
TMT: Trail making test
VREM: Short version of the Minnesota leisure time physical activity questionnaire
WAIS-IV: Wechsler adult intelligence scale
